# Factors influencing senior care and living preferences among older adults in Jiangsu, China: a cross-sectional survey study

**DOI:** 10.1186/s12913-024-11168-9

**Published:** 2024-06-12

**Authors:** Yanan Wang, Yaning Wang, Yitong Liu, Wenkun Xu, Zhuoya Yang, Zhongying Xu, Yaqin Zhong

**Affiliations:** 1 School of Public Health, Nantong University, 9 Se-yuan Road, Nantong City, Jiangsu Province 210029 China; 2 School of Medicine, Nantong University, 19 Qi-xiu Road, Nantong City, Jiangsu Province 226001 China; 3 Institute for Health Development, Nantong University, 9 Se-yuan Road, Nantong City, Jiangsu Province 210029 China

**Keywords:** Older adults, Senior care, China, Preference, Andersen behavioral model

## Abstract

**Background:**

As the population ages, senior care for older adults in China has become increasingly important and has attracted the attention of both government and society. This study aimed to explore preferences and influencing factors related to senior care among older Chinese adults and thus propose effective and targeted strategies for the development of a comprehensive care system for older adults in the aging Chinese population.

**Methods:**

Data were obtained from a cross-sectional survey conducted in sixteen communities or villages in Jiangsu Province, China, from July to September 2021. Guided by the Andersen Behavioral Model, multivariate logistic regression was conducted to identify factors associated with preferences for senior care arrangements.

**Results:**

A total of 870 respondents were included in the study, 60.11% of whom preferred receiving care in their own homes, while only 13.68% chose residential care facilities (RCFs). For predisposing factors, rural respondents preferred receiving care in their own homes compared to urban respondents (children’s home: OR = 0.55, *P* < 0.01; RCF: OR = 0.58, *P* < 0.01). Concerning enabling factors, respondents who were not employed (OR = 2.30, *P* < 0.01) and those without financial support (OR = 2.73, *P* < 0.05) preferred RCFs to their own homes. Respondents receiving life assistance (sometimes: OR = 2.76, *P* < 0.001; regularly: OR = 2.57, *P* < 0.01; every day: OR = 3.57, *P* < 0.001) preferred their children’s homes to their own homes. In terms of need factors, respondents with noncommunicable diseases (NCDs, OR > 1, *P* < 0.05), those who knew about RCFs (some: OR = 0.53, *P* < 0.005; no: OR = 0.10, *P* < 0.001) and those with a good impression of RCFs (fair: OR = 3.72, *P* < 0.05; good: OR = 11.91, *P* < 0.001) preferred receiving care in RCFs compared to their counterparts.

**Conclusions:**

Older Chinese adults’ senior care preferences were affected by predisposing factors, enabling factors, and need factors. Policy-makers should consider targeted measures to identify more precise senior care services and thus address aging challenges in China.

**Supplementary Information:**

The online version contains supplementary material available at 10.1186/s12913-024-11168-9.

## Introduction

Population aging is a crucial policy issue worldwide. According to China’s seventh census, the proportion of people aged 65 years and older was approximately 13.8% in 2020 and is predicted to reach approximately 30% by 2050 [1]. The rapidly increasing aging trend has resulted in difficulties for social and economic life in the country. The low fertility rate due to the “one-child” policy [2], changes in fertility perceptions, the irreversibility of aging, and the vulnerability of older adults in terms of physical, psychological, and social factors continue to challenge the senior care service system. Therefore, senior care has become an urgent issue in China [3].

The classification of senior care varies in China. In previous studies, three main categories of senior care have been summarized. First, according to different care responsibilities, senior care can be divided into self-support, family support, and social support [4]. Second, according to where senior care is provided, senior care can be divided into institutional senior care, community senior care, and home-based informal care [5]. Third, the modes of senior care provision can be divided into family care, state-based care, and mixed care [6]. According to previous studies, “aging in place” is the main choice among most older adults [7, 8], which is consistent with the traditional model of senior care in China. However, the mobility of young laborers has increased the number of older adults living independently, and the traditional senior care model that relies on family will not be able to meet the growing demands of older adults [9]. Therefore, preferences and influencing factors related to senior care arrangements must be explored [10] to facilitate the formulation and implementation of government policies.

China is currently undergoing a major social, economic, and demographic transition, and the senior care service sector is facing challenges. The main challenge is determining how to meet the various needs of older adults and reduce the financial burden on families and society, which is why studying the senior care and living preferences of older adults in China is particularly important. Previous studies of senior care choices have mainly focused on demographic characteristics, including gender, education level, number of children, living conditions, physical condition [11–15], and individual resources, and possible influencing factors were directly included in these analyses without a theoretical framework, resulting in limited research on senior care preferences. Senior care is a “demand-oriented” service, and studies from the demand side of living arrangements in relation to senior care can facilitate a better understanding of preferences and attitudes toward residential choices, which can help us respond to the needs of older adults and predict future needs [16, 17].

## Conceptual framework

The Andersen Behavioral Model, which guided this study, was designed to explore health service utilization [18] and has recently been extended to examine the relationship between personal choices and health service utilization among Chinese people [19]. It has been used extensively in studies of health services [20–23], mental health services [24, 25], and quality of life [26, 27], among others. The Andersen Behavioral Model distinguishes variables that contribute to different health service needs into three categories: predisposing factors, enabling factors and patients’ illness level (representing the need factor) [18, 28]. Predisposing factors include demographic and social characteristics [29]. Enabling factors refer to various resources that facilitate individual utilization of available health services [30], such as individual resources, intergenerational relationships, and social support [31, 32]. Need factors correspond to perceived or actual service and health necessities [33, 34].

Previous studies in China have indicated that residence, gender, age, marital status, and education level are predisposing factors for senior care preferences [10, 19, 29, 35]. Economic status and employment status have been defined as important indicators for measuring individual resources [36, 37]. Intergenerational relationships and social support have also been reported to be important indicators for measuring individual resources [38, 39]. Self-rated health was reported to be a subjective measure of internal perceptions and priorities [40] and has been used to evaluate a broad range of factors related to health care [41]. Furthermore, previous studies have shown that the experience of caring for older adults can influence judgments about living arrangements [42, 43]. Planning for old age has been reported to be affected by anchoring bias, which is a cognitive bias that affects decision-making [42, 44, 45]. Based on these previous findings, this study considered RCFs, including whether respondents were knowing about RCFs and their impressions of RCFs, to examine the effects described above on older adults’ senior care choices.

Although some studies have examined senior care preferences in China, most used two categories of senior care models, where cohabitation with children or living in an institution served as the dependent variable [46, 47]. Based on family structure and living arrangements in China, we used three categories (home-based care, children’s homes, and RCFs) of senior care preference as the dependent variable in the present study. The Anderson Behavioral Model was used as the theoretical framework, and potential predisposing variables constituted the baseline model. Then, enabling factors and need factors were added successively to establish Model 2 and Model 3, respectively, to evaluate the influence of predisposing factors, enabling factors and need factors and to compare the interpretation power of the three models. This study design was employed to produce holistic results and allow comparisons of the influence of each category. Based on the findings, targeted strategies were formulated to facilitate the development of a comprehensive care system for older adults in the aging Chinese population.

## Method

### Sampling and data collection

A cross-sectional survey was conducted in sixteen communities or villages in Jiangsu Province, China, from July to September 2021. These communities/villages were located in Nantong (*n* = 4751), Nanjing (*n* = 4276), Xuzhou (*n* = 4952), Changzhou (*n* = 4055), and Taizhou (*n* = 4014), which were selected based on their geographical locations and economic development. In each city, three or four communities/villages were randomly selected, and potentially eligible participants in these communities/villages were identified by rural villages or urban residential committees. Household surveys were conducted under the guidance of pertinent organizational leaders, and approximately 60 elderly people were randomly selected from each community/village. The inclusion criteria were as follows: (1) age ≥ sixty years; (2) able to understand and respond to questions asked by investigators; and (3) willingness to be interviewed. The exclusion criteria were mental disorders and poor adherence (including obviously inaccuracy answer and withdrawal from the survey process).

This study used the Raosoft online sample size calculator to calculate the lowest sample size limit with a response rate of 50%, a confidence interval of 99%, and a margin of error of 5%. The minimum sample size calculated was 645. Face-to-face questionnaire surveys (Annex 1) were conducted to collect information about the participants’ preferences for senior care. A total of 890 questionnaires were collected, and 870 valid questionnaires were ultimately obtained.

### Measurements

#### Dependent variable

The respondents’ senior care preferences were assessed using the following question: “Where do you plan to stay for your senior care?” Three response options were provided: in my own home, which was coded as 1; in my children’s home, which was coded as 2; and in a residential care facility (RCF), which was coded as 3.

#### Independent variable

Guided by the Andersen Behavioral Model and previous research, the predisposing variables included residence (urban or rural), age (60–69, 70–79 or ≥ 80), gender (male or female), marital status (married or other), and education level (elementary school and below, middle school, or high school and above).

The enabling variables included employment status (yes or no), retirement pension (yes or no), the number of children (0, 1, 2, or ≥ 3), living arrangement (living alone or not), financial support (yes or no), the frequency of life assistance (rarely or never, sometimes, regularly, or every day), the number of relatives available to meet or contact (≤ 2, 3–4 or ≥ 5), and the number of friends available to meet or contact (≤ 2, 3–4 or ≥ 5).

The need factors included self-rated health (good, fair, or poor), the number of NCDs (0, 1–2, or ≥ 3), and caring for elderly parents (yes or no). To determine participants’ opinions on senior care, they were asked, “Do you know about senior care institutions/nursing homes?” (yes, some, none) and “What is your impression of senior care institutions/nursing homes?” (poor, fair, good).

### Statistical analysis

Relationships between predisposing factors, enabling factors, need factors and senior care preferences were first assessed using chi-square tests. Then, three steps of multivariate logistic regressions were run. The dependent variable in each model was senior care preference, and the reference group was “in my own home”. In Model 1, predisposing factors were controlled. Model 2 was further adjusted for enabling factors. Model 3 was subsequently adjusted for need factors. Odds ratio (OR) with 95% confidence intervals (CIs) were used to compare the effects of different variables. The level of significance was defined as a 2-sided *P* value < 0.05. All analyses were conducted using STATA 18.0.

## Results

### Description of the sample

A total of 870 respondents aged 60 years and older were included in the study, with a response rate of 97.8%. Among the respondents, 523 (60.11%) preferred their own homes, and 13.68% preferred RCFs. The characteristics of the respondents’ predisposing factors are shown in Table 1. More than half of the respondents were rural residents (54.94%). Males accounted for 51.72% of the sample. A total of 71.15% of the respondents were married, and 28.85% were widowed. For education, 38.97% had completed high school and above. Respondents with different residences, marital statuses, and education levels had different senior care preferences (*P* < 0.05).

**Table 1:** Predisposing factors and preferences regarding senior care among the respondents (*N* = 870)

Variable	*N* (%)	Own Home (%)	Children’s Home (%)	RCF (%)	*P*
Residence					< 0.001
Urban	392(45.06)	205(52.30)	117(29.85)	70(17.86)	
Rural	478(54.94)	318(66.53)	111(23.22)	49(10.25)	
Gender					0.176
Male	450(51.72)	258(57.33)	123(27.33)	69(15.33)	
Female	420(48.28)	265(63.10)	105(25.00)	50(11.90)	
Age					0.289
60–69	392(45.06)	245(62.50)	99(25.26)	48(12.24)	
70–79	384(44.14)	229(59.64)	97(25.26)	58(15.10)	
≥ 80	94(10.80)	49(52.13)	32(34.04)	13(13.83)	
Marital status					0.005
Married	619(71.15)	393(36.49)	151(24.39)	75(12.12)	
Other	251(28.85)	130(51.79)	77(30.68)	44(17.53)	
Education level					0.003
Elementary school and below	300 (34.48)	176 (58.67)	85(28.33)	39(13.00)	
Middle school	231(26.55)	150(64.94)	64(27.71)	17(7.36)	
High school and above	339(38.97)	197(58.11)	79(23.30)	63(18.58)	

Table 2 shows the enabling factors of the respondents. Most respondents (71.15%) were not employed, and 58.28% did not have a retirement pension. A total of 1.38%, 250.57%, 41.03%, and 37.01% of the respondents with no children, one child, two children, and more than three, respectively. Furthermore, 14.94% of the respondents lived alone, and 87.28% did not receive financial support from their children. Except for the number of relatives, all other variables differed with respect to preferences for senior care (*P* < 0.05).

**Table 2:** Enabling factors and preferences regarding senior care among the respondents (*N* = 870)

Variable	*N* (%)	Own Home (%)	Children’s Home (%)	RCF (%)	*P*
Currently employed					< 0.001
Yes	251(28.85)	176 (70.12)	54(21.51)	21(8.37)	
No	619(71.15)	347(56.06)	174(28.11)	98(15.83)	
Retirement pension					0.001
Yes	363(41.72)	214(58.95)	82(22.59)	67(18.46)	
No	507(58.28)	309(60.95)	146(28.80)	52(10.26)	
Number of children					0.001
≤ 1	191(21.95)	118(61.78)	36(18.85)	37(19.37)	
2	357(41.03)	218(61.06)	91(25.49)	48(13.45)	
≥ 3	322(37.01)	187(58.07)	101(31.37)	34(10.56)	
Living arrangement					0.006
Not alone	740(85.06)	453(61.22)	198(26.76)	89(12.03)	
Alone	130(14.94)	70(53.85)	30(23.08)	30(23.08)	
Financial support^**†**^					0.024
Yes	110(12.72)	74(67.27)	30(27.27)	6(5.45)	
No	755(87.28)	449(59.47)	198(26.23)	108(14.30)	
Frequency of life assistance^**†**^					< 0.001
Rarely or never	177(20.46)	131(74.01)	23(12.99)	23(12.99)	
Sometimes (once/month)	251(29.01)	143(56.97)	69(27.49)	39(15.54)	
Regularly (≥ once/week)	235(27.17)	141(60.00)	65(27.66)	29(12.34)	
Every day	202(23.35)	108(53.47)	71(35.15)	23(11.39)	
Number of relatives					0.577
≤ 2	242(27.82)	146(60.33)	68(28.10)	28(11.57)	
3–4	334(38.39)	194(58.08)	91(27.25)	49(14.67)	
≥ 5	294(33.79)	183(62.24)	69(23.47)	42(14.29)	
Number of friends					0.004
≤ 2	295(33.91)	173 (58.64)	88(29.83)	34(11.53)	
3–4	247(28.39)	134(54.25)	77(31.17)	36(14.57)	
≥ 5	328(37.70)	216(65.85)	63(19.21)	49(14.94)	

*Notes*^**†**^ Five respondents in the study had never married and had no children

Regarding need factors, approximately 46.78% of the respondents reported good health, and 66.09% had at least one chronic disease. In terms of RCFs, 18.05% of the respondents knew about RCFs, and 14.37% reported a good impression of RCFs. As shown in Table 3, self-rated health, the number of NCDs, knowing about RCFs, and impressions of RCFs were significantly associated with the senior care preferences of the respondents (*P* < 0.05).

**Table 3:** Need factors and preferences regarding senior care among the respondents (*N* = 870)

Variable	*N* (%)	Own Home (%)	Children’s Home (%)	RCF (%)	*P*
Self-rated health					0.001
Good	407(46.78)	263(64.62)	82(20.15)	62(15.23)	
Fair	339(38.97)	200(59.00)	99(29.20)	40(11.80)	
Poor	124(14.25)	60(48.39)	47(37.90)	17(13.71)	
Number of NCDs					< 0.001
0	295(33.91)	215(72.88)	46(15.59)	34(11.53)	
1	211(24.25)	123(58.29)	57(27.01)	31(14.69)	
2	217(24.94)	113(52.07)	63(29.03)	41(18.89)	
≥ 3	147(16.90)	72(48.98)	62(42.18)	13(8.84)	
Caring for elderly parents					0.144
No	707(81.26)	418(59.12)	195(27.58)	94(13.30)	
Yes	163(18.74)	105(64.42)	33(20.25)	25(15.34)	
Knowing about RCFs					< 0.001
Yes	157(18.05)	80(50.96)	24(15.29)	53(33.76)	
Some	329(37.82)	186(56.53)	90(27.36)	53(16.11)	
None	384(44.14)	257(66.93)	114(29.69)	13(3.39)	
Impression of RCFs					< 0.001
Poor	147(16.90)	91(61.90)	51(34.69)	5(3.40)	
Fair	598(68.74)	373(62.37)	159(26.59)	66(11.04)	
Good	125(14.37)	59(47.20)	18(14.40)	48(38.40)	

#### Factors associated with senior care preferences among older adults

The multinomial logistic regression model is presented in Table 4. Living in one’s own home served as the reference category, which was compared with living in children’s homes and living in RCFs.

Among the predisposing factors, differences in residence, marital status, and education level were found to be statistically significant. Older adults living in rural areas were more inclined to choose their own homes for senior care than those living in urban areas (OR < 1). Preferences for senior care were also affected by education level; older adults at the middle school level were more likely to choose their own homes than RCFs (Model 1: OR = 0.50, *P* < 0.05; Model 2: OR = 0.48, *P* < 0.05; Model 3: OR = 0.42, *P* < 0.05).

For enabling factors, older adults who were not employed were more likely to choose RCFs (Model 2: OR = 2.08, *P* < 0.01; Model 3: OR = 2.30, *P* < 0.01) than their own homes. Those without financial support were more likely to choose RCFs than their own homes (Model 2: OR = 2.63, *P* < 0.05; Model 3: OR = 2.73, *P* < 0.05). The results also indicated that older adults were more likely to choose their children’s homes when their children more frequently provided life assistance (OR > 1). Compared with those living in children’s homes, older adults with more than five friends were more inclined to choose their own homes for senior care (Model 2: OR = 0.58, *P* < 0.01; Model 3: OR = 0.54, *P* < 0.01).

Finally, we observed that older adults with chronic diseases preferred RCFs and their children’s homes. Both knowing about RCFs (some: OR = 0.53, *P* < 0.005; none: OR = 0.10, *P* < 0.001) and impressions of RCFs (fair: OR = 3.72, *P* < 0.05; good: OR = 11.91, *P* < 0.001) were significant factors influencing senior care preferences. Respondents who knew about RCFs and those with a positive impression of RCFs preferred to RCFs for senior care.

**Table 4:** Multivariate logistic regression of senior care preferences

Independent variable	Model 1		Model 2		Model 3	
	Children’s home OR (95%CI)	RCF OR (95%CI)	Children’s home OR (95%CI)	RCF OR (95%CI)	Children’s home OR (95%CI)	RCF OR (95%CI)
**Predisposing factors**						
Location						
Urban	1.00	1.00	1.00	1.00	1.00	1.00
Rural	0.54(0.38–0.75) ***	0.49(0.31–0.76) **	0.55(0.38–0.79) **	0.57(0.35–0.92) *	0.58(0.40–0.85) **	0.55(0.32–0.96) *
Marital status						
Married	1.00	1.00	1.00	1.00	1.00	1.00
Other	0.67(0.47–0.95) *	0.52(0.34–0.81) **	0.64(0.42–0.98) *	0.76(0.42–1.38)	0.72(0.46–1.11)	0.71(0.37–1.37)
Education level						
Elementary school and below	1.00	1.00	1.00	1.00	1.00	1.00
Middle school	0.84(0.56–1.26)	0.50(0.27–0.93) *	0.97(0.64–1.49)	0.48(0.25–0.94) *	0.96(0.62–1.50)	0.42(0.20–0.88) *
High school and above	0.69(0.46–1.04)	1.23(0.74–2.03)	0.88(0.59–1.41)	1.04(0.57–1.91)	0.92(0.56–1.51)	0.87(0.44–1.71)
**Enabling factors**						
Currently employed						
Yes			1.00	1.00	1.00	1.00
No			1.33(0.91–1.94)	2.08(1.23–3.52) **	1.21(0.81–1.79)	2.30(1.28–4.14) **
Retirement pension						
Yes			1.00	1.00	1.00	1.00
No			1.39(0.92–2.11)	0.68(0.39–1.16)	1.21(0.81–1.79)	0.79(0.42–1.46)
Number of children^※^						
≤ 1			1.00	1.00	1.00	1.00
2			1.24(0.78–1.99)	0.78(0.46–1.34)	1.16(0.71–1.90)	0.78(0.43–1.43)
≥ 3			1.44(0.88–2.34)	0.66(0.37–1.19)	1.30(0.78–2.16)	0.68(0.35–1.33)
Living arrangement						
Not alone			1.00	1.00	1.00	1.00
Alone			1.41(0.80–2.50)	0.58(0.29–1.16)	1.45(0.81–2.62)	0.64(0.29–1.39)
Financial support^**†**^						
Yes			1.00	1.00	1.00	1.00
No			0.92(0.56–1.51)	2.63(1.09–6.37) *	0.89(0.53–1.47)	2.73(1.06–7.03) *
Frequency of life assistance^**†**^						
Rarely or never			1.00	1.00	1.00	1.00
Sometimes (≥ once/month)			2.56(1.48–4.43) **	1.18(0.65–2.15)	2.76(1.56–4.87) ***	1.24(0.63–2.45)
Regularly (≥ once/week)			2.37(1.36–4.14) **	0.81(0.43–1.53)	2.57(1.45–4.55) **	0.77(0.38–1.58)
Every day			3.17(1.82–5.53) ***	1.15(0.59–2.23)	3.57(2.01–6.34) ***	1.33(0.63–2.80)
Number of friends						
≤ 2			1.00	1.00	1.00	1.00
3–4			1.13(0.76–1.68)	1.42(0.82–2.48)	1.11(0.73–1.68)	0.99(0.54–1.85)
≥ 5			0.58(0.39–0.86) **	1.29(0.77–2.16)	0.54(0.35–0.82) **	0.91(0.51–1.63)
**Need factors**						
Self-rated health						
Good					1.00	1.00
Fair					1.08(0.72–1.63)	0.97(0.55–1.71)
Poor					1.38(0.77–2.48)	1.56(0.65–3.71)
Number of NCDs						
0					1.00	1.00
1					2.50(1.52–4.10) ***	2.27(1.18–4.37) *
2					2.26(1.37–3.71) **	2.58(1.33–5.01) **
≥ 3					3.17(1.81–5.55) ***	1.77(0.71–4.39)
Knowing about RCFs						
Yes					1.00	1.00
Some					1.59(0.90–2.80)	0.53(0.30–0.91) *
None					1.04(0.59–1.84)	0.10(0.05–0.21) ***
Impression of RCFs						
Poor					1.00	1.00
Fair					0.86(0.55–1.33)	3.72(1.25–11.09) *
Good					0.90(0.44–1.84)	11.91(3.70-38.39) ***
${\varvec{R}}^{2}$	0.030		0.076		0.171	

*Notes* OR: odds ratio; CI: confidence interval; Reference = own home; Significance levels: * *P* < 0.05, ** *P* < 0.01, *** *P* < 0.001; ^**†**^: five respondents in the study had never married and had no children; ^※^: older adults with no children did not choose “Children’s home”, no children and one child were combined

## Discussion

Under the analysis framework of the Andersen Behavioral Model, the present study explored preferences for and influencing factors of senior care in Jiangsu Province, China. We found considerable differences in the senior care preferences of older adults, with family care being the predominant choice, which is consistent with previous evidence [48–50].

First, older adults in different residential settings preferred their own homes, and the proportion of older adults relying on senior care institutions in rural areas was lower than that in urban areas, primarily because the addresses of senior care institutions were mostly located in cities or towns [51, 52]. We found that older adults with higher education levels were more likely to prefer living in their own homes than in senior care institutions. Studies have shown that education level influences the choice of senior care model through the associated quality of employment and life goals of older adults [29, 53]. We also found that older adults who were currently employed were more likely to prefer living in their own homes than unemployed older adults, possibly because work can provide more social interaction, which is positively associated with mental and physical health, and promote a stronger tendency toward self-care [54].

Second, intergenerational relationships and family are among the most direct sources of support for older adults [55, 56] and have a particular impact on living arrangements in Chinese culture. We found that older adults without financial support were more likely to choose RCFs, which is consistent with other studies [32]. Previous studies have confirmed that financial support can reflect the relationship between older adults and their children [57], which influences their health status and living arrangements [58]. In addition, the frequency of life assistance was also an important factor reflecting the relationship between older adults and their children [59]. Unsurprisingly, older adults were more likely to prefer living in their children’s homes when the frequency of life assistance from family was higher. Moreover, life assistance has been demonstrated to be distinct from financial support [60]; older adults who perceive more actual care and support experience greater familial intimacy and spiritual comfort [61], which can influence living arrangement choices. The results indicated that older adults with more than 3 children preferred to live in their own homes, which may suggest that care responsibilities can be shared by multiple family members in the home, thus reducing the economic burden on the children [62]. This preference was also influenced by Chinese culture [63–65], as people prefer to age at home when conditions permit.

Third, older adults with NCDs preferred to live in their children’s homes or in RCFs, suggesting that these older adults need help to maintain their lives and their health [66]. A study demonstrated that formal care services were favored by older adults, especially those with poor mental or physical conditions [67]. Interestingly, having more than 3 NCDs had a significant impact only on the preference for living in children’s homes and not on the preference to remain at home; whether this finding is attributable to the sample size in our study requires further discussion. We also found that the respondents who did not know about RCFs tended to live in their own homes rather than in RCFs. A plausible explanation could be that decision-making is influenced by the degree of information mastery [68], and a more familiar environment provides security and independence [69]. In addition, older adults with a good impression of RCFs were more receptive to institutional care. Favorable exposure to RCFs has been reported to be associated with a positive attitude toward RCFs [70]. According to First Impress [71], older adults’ impressions of RCFs directly affect their choice of senior care, and older adults with positive impressions prefer RCFs [5]. Notably, few respondents preferred RCFs in the present study, and our results indicated that most respondents had little knowledge about RCFs. To develop RCFs, policy-makers should strengthen publicity among older adults.

### Strength

The strength of this study was our use of the Andersen Behavioral Model to investigate senior care preferences among older adults. We added potential influencing factors to the model, including individual resources, intergenerational relationships, family support and social support, as well as extended need factors, including knowing about and impressions of RCFs, to explore their influence on care choices. Previous research has demonstrated that older adults prefer to live in their own homes for as long as possible [72]. Nevertheless, with the transformation of the Chinese family structure, the perception of traditional senior care has markedly changed, the concept of independent senior care has gradually emerged, and more attention has been directed toward institutional care [46]. Moreover, as shown in the results, having a good impression of RCFs and knowing about RCFs may increase older adults’ willingness to live in such institutions, which needs to be considered. Overall, receiving senior care from professional institutions is desirable for older adults. More specifically, the transition from family care to socialized care will become an inevitable trend in the future.

Our study provides three primary contributions. First, we defined three categories of living arrangements for older adults’ senior care preferences by asking participants where they preferred to live as they continued to age. Expanding the understanding of older adults’ senior care preferences and attitudes is critical for forecasting and responding to demands for senior care [73]. Second, we comprehensively integrated, expanded, and analyzed factors affecting the senior care preferences of older adults based on the Andersen Behavioral Model, including psychosocial factors, individual resources, intergenerational relationships, family and social support, and knowing about and impressions of senior care institutions. Third, we analyzed older adults’ preferences through their choice of living arrangement as they age, which enriched the existing research on the preferences of older adults. Although family care predominated among the senior care preferences of the surveyed older adults, institutional care was gradually accepted by some older adults, which may soon become a better choice for China [6].

### Policy implications

According to the findings for senior care preferences among older adults in our study, meeting older adults’ needs required not only a change in people’s conceptions of senior care but also strong support from the government and consideration from society. First, urban and rural senior care systems should be constructed based on the needs of older adults and improved to provide targeted senior care services. Second, different senior care models urgently need to be promoted through the internet, television, newspapers, and other media, and participation from multiple parties and social interactions should be encouraged. Third, laws regarding the senior care service system should be updated considering different senior care models, and high-quality services should be provided under third-party supervision.

### Limitations

This study had several limitations that need to be addressed. First, the respondents were recruited from several cities in Jiangsu Province; therefore, the generalizability of the study findings is limited. Future studies should include larger and more diverse samples to increase representativeness. The classification and inclusion criteria for variables based on the Andersen Behavioral Model may vary in different studies. Additionally, the survey used in this study included some recall questions, and the responses may have been biased due to the aging of the older adults, who may have had poor memories and cognitive impairment. Finally, since our data were collected at one point in time, no temporal changes in participants’ preferences for senior care could be measured. Studies have shown that willingness and preferences for senior care usually change over time [48]. Future studies should further investigate changes in older adults’ senior care preferences over time.

## Conclusions

Our study found that preferences for senior care among older adults were affected by predisposing factors, enabling factors, and need factors. China is in a critical initial period of transition with respect to pensions, and the results of this study will provide new perspectives for policy-makers when addressing the challenges faced by older adults in China. This study can serve as a reference for the allocation of senior care resources to meet the growing needs of older adults.

### Electronic supplementary material

Below is the link to the electronic supplementary material.

Supplementary Material 1

Supplementary Material 2

Supplementary Material 3

## Data Availability

The datasets used and/or analyzed during the current study are available from the corresponding author upon reasonable request.
