# Supplementary material for: Factors influencing senior care and living preferences among older adults in Jiangsu, China: a cross-sectional survey study
Source: BMC Health Serv Res. 2024 Jun 12;24:723. doi: 10.1186/s12913-024-11168-9 (PMC11167893; doi:10.1186/s12913-024-11168-9)
Supplement: Supplementary file 1 — Supplementary Material 1 [file 12913_2024_11168_MOESM1_ESM.docx]

**Survey on the Health Status and senior care of older adults**

We are members of the research group "Health status and senior care of old adults" of Nantong University. To understand the health status and senior care of older adults, we conducted this survey. This survey is anonymous, and your information is only used for research analysis. Thank you for your cooperation!

**A. Basic information**

|  | **Questions and choices** |  |
| --- | --- | --- |
| **A01** | **Place of investigation:**  ①Urban ②Rural |  |
| **A02** | **Gender:** ①Male ②Female |  |
| **A03** | **Age:** |  |
| **A04** | **What is your marital status?**  ①Married with a spouse ②Widowed ③Divorced ④Unmarried |  |
| **A05** | **What is your education?**  ①Illiterate ②Sishu/home school ③Elementary school ④Middle school  ⑤high school/vocational school ⑥Two-/Three-Year College/Associate degree  ⑦Bachelor's degree and above |  |
| **A06** | **Are you currently working?**  ①Yes ②No (**skip to A08**) |  |
| **A07** | **What is your current job?**  ①Leaders of the state, enterprises and institutions ②Professional and technical staff  ③General office staff ④General workers in commerce/service/manufacturing  ⑤Self-employed, freelancers ⑥Farmers, herders, fishermen ⑦Others |  |
| **A08** | **What is your job before you stopped working?**  ①Leaders of the state, enterprises and institutions ②Professional and technical staff  ③General office staff ④General workers in commerce/service/manufacturing  ⑤Self-employed, freelancers ⑥Farmers, herders, fishermen ⑦Others |  |
| **A09** | **Do you have a pension?** ①Yes ②No |  |
| **A10** | **What is your current monthly income?**  CNY |  |
| **A11** | **How many houses do you and your spouse have?**  ①No ②One ③Two or three ④More than three |  |
| **A12** | **How do you feel about your family’s financial situation?**  ①Very good ②Good ③Average ④Not good ⑤Very bad |  |
| **A13** | **How many sons do you have?** |  |
| **A14** | **How many daughters do you have?** |  |
| **A15** | **How many people (including yourself) live with you?**  **(If living alone, skip to A15)** |  |
| **A14** | **Who are them? (Multiple choice)**  (1)Spouse (2)Unmarried partner (3)Son (4)Daughter (5)Parent (6)Spouse's parent (7)Sibling (8)Son-in-law (9)Daughter-in-law (10)grandchild or his/her spouse  (11)Nanny (12)Other |  |
| **A15** | **Are you satisfied with your life?**  ①very satisfied ②satisfied ③fair ④dissatisfied ⑤very dissatisfied |  |

**B. Health status**

| **B01** | **What’s your self-rated health?**  ①Very good ②Good ③Average ④Not good ⑤Very bad |  |
| --- | --- | --- |
| **B02** | **Are you currently suffering from a chronic disease?** ①Yes ②No (**skip to section C**) |  |
| **B03** | **What chronic conditions do you suffer from? (Multiple answers possible)**  (1) Hypertension (2) Heart disease/coronary heart disease (3) Diabetes mellitus  (4) Cerebrovascular disease (including stroke) (5) Kidney disease (6) Liver disease  (7) Tuberculosis (8) Rheumatoid (9) Cervical/lumbar diseases (10) Arthritis (11) Breast diseases (12) Reproductive system diseases (13) Prostate diseases (14) Urological Diseases  (15) Glaucoma/Cataracts (16) Cancer/Malignant Tumors (17) Alzheimer's Disease  (18) Osteoporosis (19) Chronic bronchitis/other respiratory diseases (20) Neurological diseases (21) Gastroenteritis or other digestive diseases (22) Parkinson's disease (23) Deafness  (24) Other chronic diseases |  |

**C. Activities of daily living**

|  |  | No difficulty Difficult but can do Need help Can't do it |  |
| --- | --- | --- | --- |
| **C01** | Dress | ① ② ③ ④ |  |
| **C02** | Take a bath | ① ② ③ ④ |  |
| **C03** | Eat | ① ② ③ ④ |  |
| **C04** | Getting into/out of bed | ① ② ③ ④ |  |
| **C05** | Use the toilet | ① ② ③ ④ |  |
| **C06** | Control urination and defecation | ① ② ③ ④ |  |

**D. Senior Care and Living Preferences**

| **D01** | **Do you need to care for elderly parents including your own parents or Spouses’ parents?**  ①Yes ②No**(skip to D03)** |  |
| --- | --- | --- |
| **D02** | **In the past month, how long had you (or your spouse) average spend in caring for elderly parents in one week？**  Hours |  |
| **D03** | **Where do you plan** **to stay for your senior care?**  ①Own home ②Children's home ③Residential care facility |  |
| **D04** | **Do you know about residential care facilities (senior care institutions/nursing homes)?**  ①Yes ②Some ③No |  |
| **D05** | **In general, what is your impression of senior care institutions/nursing homes?**  ①Poor ②Fair ③Good |  |
| **D06** | **Which circumstances would you choose residential care facilities (senior care institutions/nursing homes)? (Multiple answers possible)**  ①In poor health and in need of care ②Lonely and in need of company  ③Have a family conflicts ④Change living environment ⑤Reduce the burden on children ⑥Others ⑦Never |  |
| **D07** | **If you need to live in residential care facilities (senior care institutions/nursing homes), how much can your family afford a month?**  CNY |  |

**E. Intergenerational relationship**

| **E01** | **In general, how is your relationship with your child?**  ①Very good ②Good ③Average ④Not good ⑤Very bad |  |
| --- | --- | --- |
| **E02** | **Do you have a financial support from your child in past 12 months？**  ①No ②Yes**(skip to E03)** |  |
| **E03** | **How much money has the child give you in past 12 months？**  CNY |  |
| **E04** | **How many times has the child helped you for life assistance (such as household chores) in past 12 months?**  ①Almost every day ②At least once a week ③At least once a month  ④Once every few months ⑤Never contact |  |
| **E05** | **How many times has you take care of grandchildren in past 12 months?**  ①Almost every day ②At least once a week ③At least once a month  ④Once every few months ⑤Never contact |  |
| **E06** | **Do you think you are emotionally close to your children?**  ①Usually ②Often ③Sometimes ④Never |  |
| **E07** | **Do you think that your children are not sufficiently concerned about you?**  ①Usually ②Often ③Sometimes ④Never |  |

**F. Social support**

|  |  | 0 1 2 3-4 5-8 9+ |  |
| --- | --- | --- | --- |
| **F01** | How many families/relatives do you meet or contact at least in one month? | ① ② ③ ④ ⑤ ⑥ |  |
| **F02** | How many families/relatives do you think you can talk to about your privacy? | ① ② ③ ④ ⑤ ⑥ |  |
| **F03** | How many families/relatives can help you when you are in need? | ① ② ③ ④ ⑤ ⑥ |  |
| **F04** | How many friends do you meet or contact at least in one month? | ① ② ③ ④ ⑤ ⑥ |  |
| **F05** | How many friends do you think you can talk to about your privacy? | ① ② ③ ④ ⑤ ⑥ |  |
| **F06** | How many friends can help you when you are in need? | ① ② ③ ④ ⑤ ⑥ |  |
