# Supplementary material for: Factors influencing senior care and living preferences among older adults in Jiangsu, China: a cross-sectional survey study
Source: BMC Health Serv Res. 2024 Jun 12;24:723. doi: 10.1186/s12913-024-11168-9 (PMC11167893; doi:10.1186/s12913-024-11168-9)
Supplement: Supplementary file 2 — Supplementary Material 2 [file 12913_2024_11168_MOESM2_ESM.docx]

**Declarations**

Ethics approval and consent to participate

Ethical approval was received from the Institutional Review Boards at the University of Nantong. All participants gave informed consent before participating in the study and informed consent was obtained from their legal guardian/patents. This study was conducted in accordance with the relevant guidelines and regulations.

**Consent for publication**

Not applicable.

**Competing interests**

No potential conflict of interest was reported by the authors.

**Authors’ contributions**

Yanan Wang and Yaning Wang carried out the study and holds the main responsibility for writing the manuscript, as well as Yanan Wang and Yaning Wang revised the manuscript. Yitong Liu and Wenkun Xu conducted the statistical analysis. Zhuoya Yang and Zhongying Xu drafted parts of the paper and revised the manuscript. Yanan Wang and Yaqin Zhong contributed equally to this work. All authors provided input during the preparation of the manuscript and approved the final version.

**Availability of data**

The datasets used and/or analyzed during the current study are available from the corresponding author upon reasonable request

**Funding**

This work was supported by grants from Humanities and Social Science Foundation of the Ministry of Education in China (Number: 21YJA840018), National Social Science Foundation (Number: 23BSH151), and Funding of Nantong Science and Technology Program (MS2023084). The granting agencies did not have roles in the design, collection, analysis, and interpretation of data or in writing the manuscript.
