# Supplementary material for: Factors influencing senior care and living preferences among older adults in Jiangsu, China: a cross-sectional survey study
Source: BMC Health Serv Res. 2024 Jun 12;24:723. doi: 10.1186/s12913-024-11168-9 (PMC11167893; doi:10.1186/s12913-024-11168-9)
Supplement: Supplementary file 3 — Supplementary Material 3 [file 12913_2024_11168_MOESM3_ESM.docx]

**Factors Influencing Senior Care and Living Preferences Among Older Adults in Jiangsu, China: A cross-sectional survey study**

Yanan Wang^1†^, Yaning Wang^1†^, Yitong Liu^1^, Wenkun Xu^1^, Zhuoya Yang^1^, Zhongying Xu^2^, Yaqin Zhong^1*^

^1^ School of Public Health, Nantong University, 9 Se-yuan Road, Nantong City, 210029, Jiangsu Province, China

^2^ School of Medicine, Nantong University, 19 Qi-xiu Road, Nantong City, 226001, Jiangsu Province, China

^†^ These authors have contributed equally to this work

Yanan Wang Email: [Yanan997447@163.com](mailto:Yanan997447@163.com)

Yaning Wang Email: [wyn2000528@163.com](mailto:wyn2000528@163.com)

Yitong Liu Email: [strawberry_nana121@163.com](mailto:strawberry_nana121@163.com)

Wenkun Xu Email: [xuwenkun10@163.com](mailto:xuwenkun10@163.com)

Zhuoya Yang Email: [17843305379@163.com](mailto:17843305379@163.com)

Zhongying Xu Email: 3069744051@qq.com

Yaqin Zhong Email: [yqzhong@ntu.edu.cn](mailto:yqzhong@ntu.edu.cn)

***Corresponding author**: Yaqin Zhong

School of Public Health, Nantong University, 9 Se-yuan Road, Nantong City, 210029, Jiangsu Province, China

**ORCID iD**

Yaqin Zhong  <https://orcid.org/0000-0001-9467-7239>
